# Prevalence of coronary artery calcification and its association with mortality, cardiovascular events in patients with chronic kidney disease: a systematic review and meta-analysis

**DOI:** 10.1080/0886022X.2019.1595646

**Published:** 2019-04-24

**Authors:** Xue-Rong Wang, Jing-Jing Zhang, Xing-Xin Xu, Yong-Gui Wu

**Affiliations:** a Department of Nephrology, The First Affiliated Hospital of Anhui Medical University, Hefei, China;; b Department of Nephrology, The Second Hospital of Anhui Medical University, Hefei, China

**Keywords:** Chronic kidney disease, coronary artery calcification, cardiovascular mortality, all-cause mortality, cardiovascular events

## Abstract

**Purpose:** To date, the prevalence and prognostic role of coronary artery calcification (CAC) in patients with chronic kidney disease (CKD) have been investigated in several studies, but have yielded conflicting results. The aim of this meta-analysis is to derive a more precise estimation of CAC prevalence in CKD patients and its association with cardiovascular events and mortality.

**Methods:** The relevant literature was identified and evaluated from inception until July 2018 through multiple search strategies on PubMed, Embase, and Web of Science. Cross-sectional or cohort (baseline data) studies reporting CAC prevalence were included. Data extracted from eligible studies were used to calculate effect estimates (ESs) and 95% confidence intervals (95%CI). We searched databases for observational studies that explored baseline CAC and subsequent cardiovascular or all-cause mortality risk in CKD patients.

**Results:** The meta-analysis included 47 studies; 38 of these were included in the final analysis of CAC prevalence. The pooled prevalence of CAC in random effect model was 60% (95%CI 53–68%). CAC was positively associated with an increased risk of all-cause mortality (hazard ratio [HR] 3.44; 95%CI 2.40–4.94), cardiovascular mortality (HR 3.87; 95%CI 2.06–7.26), and cardiovascular events (HR 2.09; 95%CI 1.19–3.67), when comparing individuals in the top CAC score group to those in the bottom CAC score group.

**Conclusions:** The pooled prevalence of CAC is highly prevalent. CAC is independently associated with all-cause and cardiovascular mortality risk as well as cardiovascular events among CKD patients. In view of the high heterogeneity, larger clinical trials are still needed.

## Introduction

Deterioration in renal function is associated with marked increase in cardiovascular mortality. More than 50% of deaths in patients with end stage renal disease (ESRD) are attributable to cardiovascular disease [1]; patients with chronic kidney disease (CKD) are at higher risk of coronary artery calcification (CAC). CAC is known to be associated with cardiovascular events as well as cardiovascular and all-cause mortality [2–5]. However, another study found that vascular calcification did not independently predict mortality in predialysis patients [6]. Emerging studies found that CAC was common in predialysis patients [7]; another study showed that CAC was more prevalent in dialysis patients, up to 93% [8].

CAC can be effectively quantified using electron-beam or multi-detector computed tomography (EBCT/MDCT), by measuring total calcium levels. CAC among CKD patients is defined as an Agatston score >0. Uremia-related risk factors, such as higher plasma calcium and phosphate, as well as increased oxidative stress, contribute to CAC progression.

CAC is present in early CKD patients, but is more common in dialysis patients. Better understanding of the epidemiology of CAC in CKD populations would potentially contribute to the formulation of strategies in further clinical intervention. Although several studies have reported positive association between CAC and CKD, the prevalence of CAC varies considerably. The results of these studies were inconsistent, which may be due to inadequate statistical power, publication bias, ethnic differences or uncorrected multiple hypothesis testing. Therefore, to overcome the limitations of individual studies, a meta-analysis was performed to overcome the limitations of individual studies, to explore the prevalence of CAC among predialysis, dialysis, and renal transplant patients along with studying the impact of CAC on clinical outcomes.

## Methods

The systematic review and meta-analysis were performed according to the Meta-analysis of Observational Studies in Epidemiology (MOOSE) guidelines [9].

### Search strategy

A comprehensive search was carried out in PubMed, Embase, and Web of Science databases from inception of the study until July 2018. The search terms used were as follows: ‘coronary artery calcification’ or ‘vascular calcification’ and ‘chronic kidney disease’ or ‘chronic renal failure’ or ‘chronic kidney failure’ or ‘hemodialysis’ or ‘peritoneal dialysis’ or ‘uremia’ and ‘death’ or ‘mortality’. The search keywords were searched both as medical subject headings (MeSH) and text words, without restrictions on ethnicity or geographic area. References of included studies were searched for eligible articles.

### Inclusion and exclusion criteria

Articles were included when they met the following criteria: (1) observational studies (cross-sectional or cohort study); (2) studies with a clear definition of CKD; (3) CAC was assessed using a calcification score; (4) the studies provided the prevalence of CAC, or sufficient data to calculate it. Exclusion criteria were as follows: (1) comments, review articles, meetings, letters, case reports, meta-analyses and unrelated or animal studies. (2) Young patients (less than 18 years old). (3) Containing insufficient data.

### Data extraction and quality assessment

According to the MOOSE guidelines, we attempted to extract the following information: first author name, publishing year, ethnicity, study type, point prevalence, sample size, age, percentage of men, dialysis duration, hazard ratio (HR) and 95%CI of mortality and cardiovascular events. Data of selected studies were independently extracted by two reviewers (X.W. and X.X.). In case of disagreement, the issue was resolved by a third investigator (J.Zh.). When original important data were missing, we contacted corresponding authors to obtain the relevant data. We did not impute missing data.

The quality of included studies was independently assessed by the two reviewers (X.W. and X.X.), using a special assessment [10,11]. Studies with a score of 0–6 were considered as poor quality, and those with a score of 7–13 were considered as high quality. A set of 13 criteria (study attrition, study participation, outcome measurement, confounding measurement, and analysis) was predefined.

### Statistical analysis

We used *I*^2^ statistic to identify heterogeneity. If *I*^2^ ≥ 50% or *p* < 0.05, a random effect model was applied; otherwise, a fixed effect model was used. In addition, low, moderate, and high levels were nominally applied to define *I*^2^ values as 25%, 50%, and 75%, respectively [12]. In addition, subgroup analyses were conducted when the heterogeneity was high. We conducted univariate meta-regression analysis to explore the effects of disease-related covariates on the CAC prevalence estimates.

HR and 95%CI were extracted from studies that reported proportional hazard regression, and a natural logarithm scale was conducted.

Begg’s test and funnel plots were performed to evaluate publication bias. *p* Values of Begg’s test <0.05 and asymmetry of funnel plots showed the possibility of publication bias.

Finally, *p* value <0.05 was considered to be significant difference. Statistical analyses for this article were conducted using STATA version 12.0 (Stata Corporation, College Station, TX).

The data of kappa of agreement during the literature search were analyzed using SPSS Statistics version 17.0 (SPSS Inc., Chicago, IL). The data of kappa >0.60 were considered to indicate good agreement.

## Results

### Study selection and quality assessment

**Figure 1.**
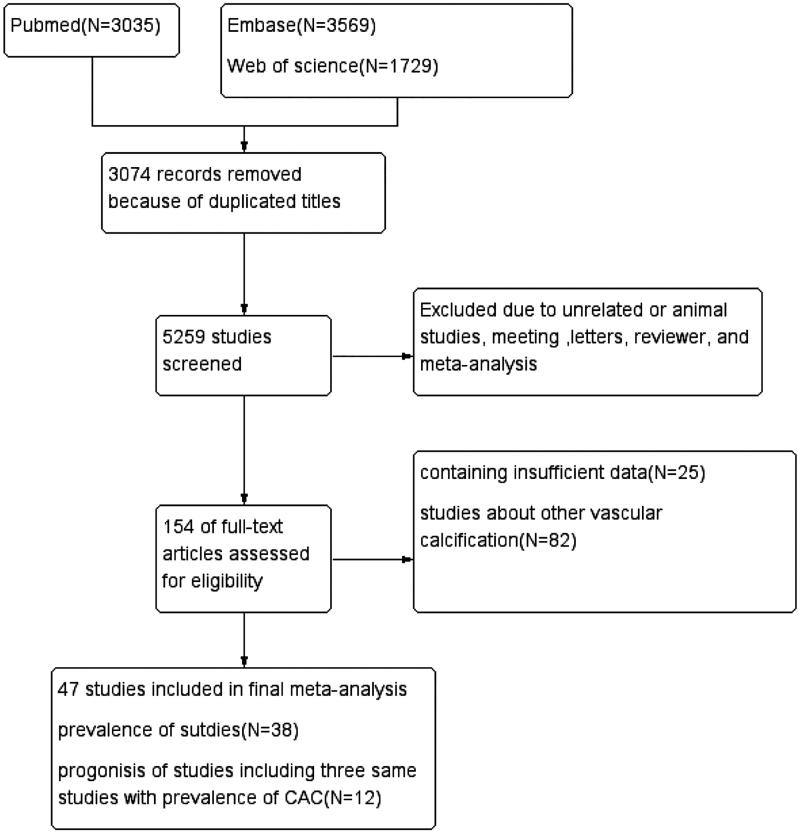
Flowchart of the study selection process.

The flowchart of study selection for inclusion and exclusion is presented in Figure 1. Of the eligible 38 CAC prevalence studies [7,8,13–48], there were 25 cross-sectional studies, 13 cohort studies in the final analysis, as seen in Table 1. The mean age of the participants ranged from 48 to 68.7 years. The articles included 17 predialysis, two renal transplantation, and 19 dialysis studies. The reported diagnostic methods for CAC were uniform across all the included studies. Among the publications, 37 studies were in English and one was in Chinese.

**Table 1.** Characteristics of included articles in CAC prevalence.

Reference	Region	Design	Sample	CKD stage	Diagnostic method	Prevalence
Krajnc et al. [17]	European	Cross-sectional	45	Hemodialysis	Agatston score	0.24
Suh-Chiou et al. [14]	South America	Cross-sectional	4189	Predialysis	Agatston score	0.28
Russo et al. [7]	European	Cross-sectional	85	Predialysis	Agatston score	0.40
Bae et al. [15]	Asia	Cross-sectional	423	Hemodialysis	Agatston score	0.64
Freercks et al. [16]	South Africa	Cross-sectional	75	Dialysis	Agatston score	0.38
Nitta et al. [8]	Asia	Cross-sectional	53	Hemodialysis	Agatston score	0.93
Machado et al. [18]	South America	Cross-sectional	373	Predialysis	Agatston score	0.79
Abdelmalek et al. [19]	North America	Cohort study	93	Hemodialysis	Agatston score	0.25
Garland et al. [20]	North America	Cross-sectional	125	Predialysis	Agatston score	0.86
Russo et al. [21]	Asia	Cohort study	341	Predialysis	Agatston score	0.40
Chen et al. [22]	Asia	Cohort study	1541	Predialysis	Agatston score	0.60
Rosas et al. [23]	North America	Cross-sectional	79	Renal transplant	Agatston score	0.63
Di Iorio et al. [24]	North America	Cohort study	132	Hemodialysis	Agatston score	0.71
Bonifacio et al. [25]	North America	Cohort study	41	Hemodialysis	Agatston score	0.46
Cianciolo et al. [26]	European	Cross-sectional	253	Hemodialysis	Agatston score	0.90
Porter et al. [48]	Asia	Cross-sectional	112	Hemodialysis	Agatston score	0.60
Srivaths et al. [27]	North America	Cross-sectional	16	Hemodialysis	Agatston score	0.31
Stavroulopoulos et al. [28]	European	Cohort study	103	Predialysis	Agatston score	0.59
Bargnoux et al. [29]	European	Cohort study	83	Renal transplant	Agatston score	0.39
Kim et al. [46]	Asia	Cross-sectional	470	Predialysis	Agatston score	0.34
Garland et al. [31]	North America	Cohort study	119	Predialysis	Agatston score	0.83
Liu et al. [30]	Asia	Cross-sectional	1423	Dialysis	Agatston score	0.68
Sevinc Ok et al. [32]	Asia	Cross-sectional	50	Peritoneal dialysis	Agatston score	0.52
Budoff et al. [33]	North America	Cross-sectional	1908	Predialysis	Agatston score	0.65
Cui et al. [34]	Asia	Cross-sectional	53	Hemodialysis	Agatston score	0.76
Asci et al. [35]	North America	Cross-sectional	207	Hemodialysis	Agatston score	0.69
Chang et al. [36]	Asia	Cross-sectional	870	Predialysis	Agatston score	0.67
Patsalas et al. [37]	European	Cross-sectional	40	Hemodialysis	Agatston score	0.58
Kurnatowska et al. [38]	European	Cross-sectional	47	Hemodialysis	Agatston score	0.70
Shantouf et al. [39]	North America	Cohort study	166	Hemodialysis	Agatston score	0.89
Bundy et al. [42]	North America	Cohort study	1123	Predialysis	Agatston score	0.61
Nishizawa et al. [13]	Asia	Cross-sectional	207	Hemodialysis	Agatston score	0.93
Mehrotra et al. [43]	North America	Cross-sectional	60	Predialysis	Agatston score	0.93
Kestenbaum et al. [44]	North America	Cohort study	562	Predialysis	Agatston score	0.66
Koukoulaki et al. [45]	European	Cross-sectional	49	Predialysis	Agatston score	0.79
McPherson et al. [40]	North America	Cohort study	721	Predialysis	Agatston score	0.35
Tuttle and Short [41]	North America	Cohort study	883	Predialysis	Agatston score	0.28
Janicka et al. [47]	European	Cross-sectional	102	Peritoneal dialysis	Agatston score	0.66

The predialysis stages include CKD 1–5 stages.

The average score of quality assessment was 8.71 in CAC prevalence studies. Of the 38 prevalence studies, nine studies were considered poor quality. In 12 CAC prognosis articles (Table 2), the average score of quality assessment was 9.46, and only two studies were of poor quality. The data of kappa of agreement during quality assessment were 0.747, indicating good agreement.

**Table 2.** Characteristics of the included articles on CAC prognosis.

References	Country	Design	Sample size	CKD stage	Diagnostic method	HR (95%CI)
Abdelmalek et al. [19]	USA	Cohort study	93	Hemodialysis	Agatston score	A: all-cause mortality 2.86 (1.24–6.6)
						A: cardiovascular mortality 2.41 (1.04–5.59)
						A: cardiovascular event 1.7 (0.4–7.3)
Chen et al. [22]	New Orleans	Cohort study	1541	Predialysis	Agatston score	A: all-cause mortality 1.42 (0.82–2.46)
						B: all-cause mortality 1.59 (1.17–2.18)
						A: cardiovascular event 1.91 (0.85–4.27)
						B: cardiovascular event 1.44 (1.02–2.02)
Shantouf et al. [39]	USA	Cohort study	166	Hemodialysis	Agatston score	A: all-cause mortality 13.3 (1.3–65.1)
Wilkieson et al. [49]	Canadian	Cohort study	248	Hemodialysis	Agatston score	A: all-cause mortality 2.4 (0.45–12.97)
Chiu et al. [50]	USA	Cohort study	225	Predialysis	Agatston score	A: all-cause mortality 3.54 (1.61–7.77)
Nguyen et al. [51]	Belgium	Cohort study	281	Renal transplantation	Agatston score	B: cardiovascular event 1.4 (1.12–1.75)
Fensterseifer et al. [52]	Brazil	Cohort study	59	Hemodialysis	Agatston score	A: all-cause mortality 3.53 (0.71–17.43)
Hwang et al. [53]	Korea	Cohort study	30,703	Predialysis	Agatston score	A: all-cause mortality 2.86 (2.209–3.702)
Russo et al. [54]	Italy	Cohort study	181	Predialysis	Agatston score	A: cardiovascular event 8.4 (2.3–30.1)
Yan et al. [55]	China	Cohort study	254	Peritoneal dialysis	Agatston score	A: all-cause mortality 6.43 (3.86–10.72)
						A: cardiovascular mortality 7.087 (2.74–18.37)
						A: cardiovascular event 4.27 (2.09–8.29)
Liu et al. [56]	China	Cohort study	1493	Dialysis	Agatston score	A: all-cause mortality 4.15 (2.08–8.27)
Zhe et al. [57]	China	Cohort study	86	Hemodialysis	Agatston score	A: all-cause mortality 7.68 (1.69–34.82)

A for hazard ratio (the highest CAC score vs. the lowest CAC score), B for hazard ratio (on a natural log scale), HR hazard ratio, 95%CI 95% confidence intervals.

### CAC prevalence

**Figure 2.**
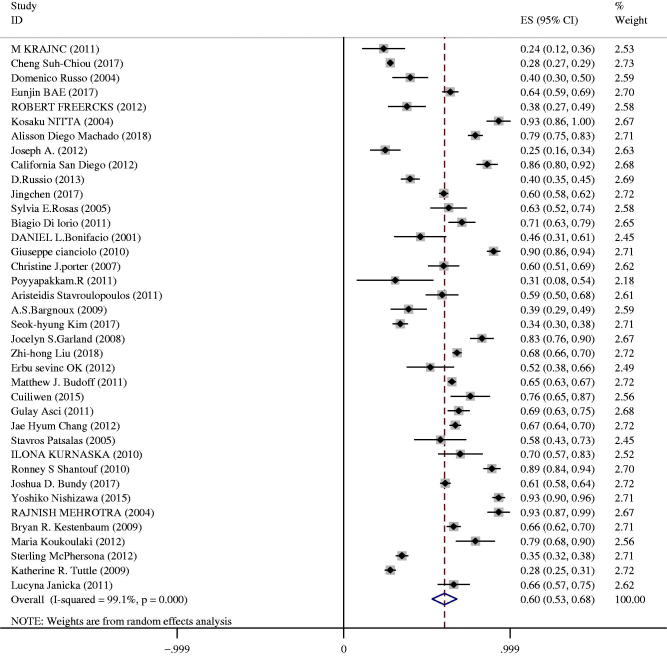
Forest plot of prevalence estimates of CAC in CKD patients.

As shown in Figure 2, the overall prevalence of CAC among CKD patients was 60% (95%CI 53–68%). Significant heterogeneity was observed in the meta-analysis (*I*^2^ = 99%, *p* < 0.01). A random effect model was applied since the heterogeneity was high.

**Figure 3.**
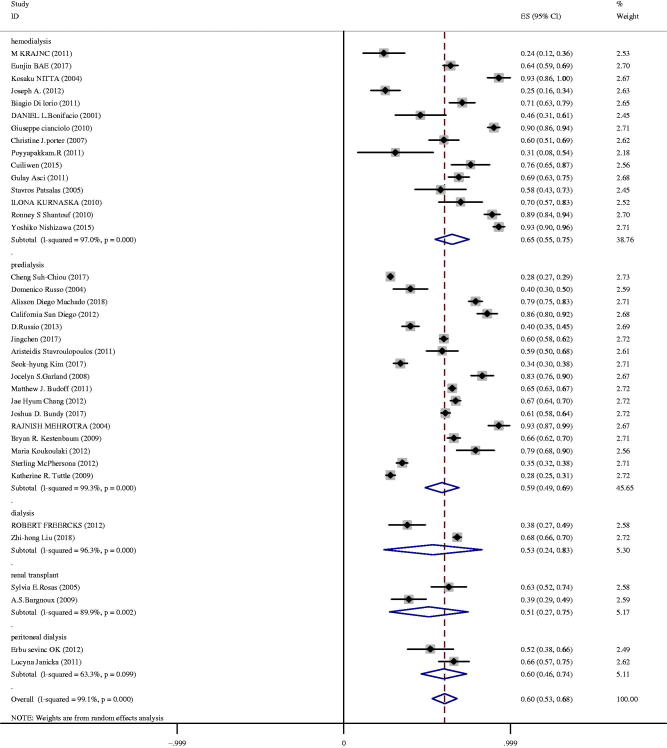
Prevalence of CAC in patients with different CKD stages.

In the predialysis patients, prevalence of CAC ranged from 28 to 93%, and the pooled prevalence was 59% (95%CI 49–69%) with high heterogeneity (*I*^2^ = 99%) (Figure 3). The prevalence of CAC in patients with hemodialysis ranged from 24 to 93%, and the pooled prevalence was 65% (95%CI 55–75%). In the setting of renal transplantation, the pooled prevalence was 51% (95%CI 27–75%). The prevalence of CAC in patients with peritoneal dialysis ranged from 52 to 66%, and the pooled prevalence was 60% (95%CI 46–74%). The pooled prevalence was 53% (95%CI 24–83%) in patients at advanced stages, who received hemodialysis and peritoneal dialysis. A random effect model was used in the analysis, as the heterogeneity was high.

### Subgroup analysis and meta-regression analysis

In most cases, significant heterogeneity was observed, so we performed a subgroup analysis. The pooled estimates of CAC prevalence in different subgroups are shown in Table 3. A significant difference was found between older age (≥60 years) and younger age (<60 years).

**Table 3.** CAC prevalence in different subgroups.

Subgroup	No of studies	Prevalence	95%CI	*I*^2^ (%)	*p*^a^	*p*^b^
Study design						<0.001
Cross-section	25	0.54	0.43–0.65	98.6	<0.001	
Cohort study	13	0.64	0.54–0.74	99.3	<0.001	
Country						<0.001
Asia	11	0.64	0.54–0.74	98.4	<0.001	
Non-Asia	27	0.59	0.49–0.68	99.2	<0.001	
Age						<0.001
≥60 years	12	0.67	0.56–0.78	97.8	<0.001	
<60 years	26	0.57	0.49–0.66	99.1	<0.001	
CKD stage						<0.001
Predialysis	17	0.59	0.49–0.69	99.3	<0.001	
Dialysis/renal transplant	21	0.62	0.54–0.70	96.8	<0.001	
Modality of dialysis	17					<0.001
Hemodialysis	15	0.65	0.55–0.75	97	<0.001	
Peritoneal dialysis	2	0.60	0.46–0.74	63.3	0.09	
Sample size						<0.001
<200	22	0.59	0.49–0.69	96.3	<0.001	
≥200	16	0.62	0.51–0.72	99.6	<0.001	
Study published						<0.001
Before 2010	14	0.66	0.53–0.68	98.8	<0.001	
2010–2018	24	0.57	0.49–0.66	99.2	<0.001	

^a^*p* Value for heterogeneity among studies in each group.

^b^*p* Value for interaction evaluated between subgroups.

**Figure 4.**
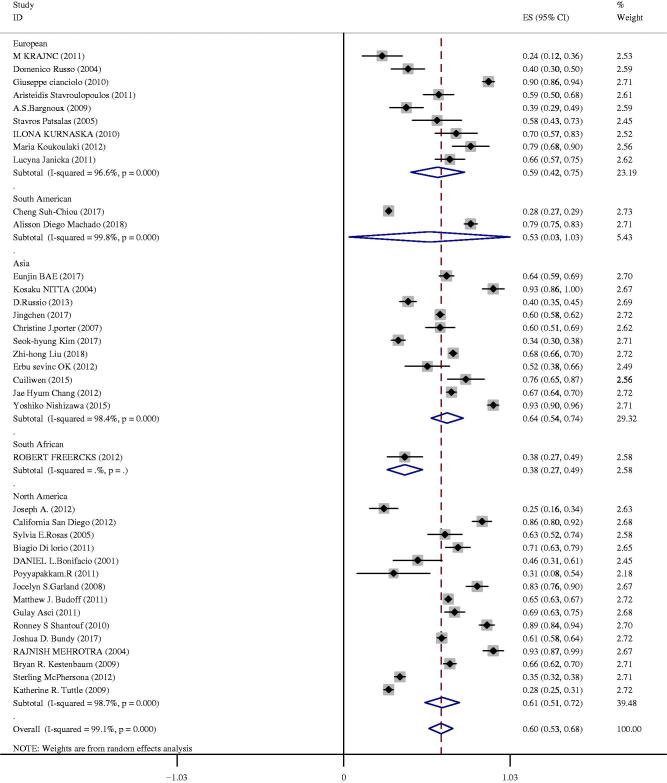
Prevalence of CAC among CKD patients in different regions.

Geographical differences in CAC prevalence among CKD patients were observed (in Figure 4). Significant heterogeneity was observed in the meta-analysis (*I^2^* = 99.1%, *p* <0 .01), so we conducted a random effect model. Patients in Asia presented with the highest rates 64% (95%CI 54–74%), followed by North America 61% (95%CI 51–72%), European 59% (95%CI 42–75%), South America 53% (95%CI 3–103%). The lowest rate of CAC prevalence was found in South Africa 38% (95%CI 27–49%). However, only one study was performed in South Africa, which may be responsible for the heterogeneity. The prevalence of CAC in Asia was 64% (95%CI 54–74%), the pooled prevalence in other countries was 59% (95%CI 49–68%), a significant difference was found in the subgroup analysis (*p* < 0.001).

Subgroup analysis based on the variable (predialysis participants, dialysis and renal replacement therapy participants) was the source of heterogeneity (*p* < 0.001). Subgroup analysis of prevalence based on modality of dialysis (hemodialysis vs. peritoneal dialysis) contributed to the source of heterogeneity (*p* < 0.001).

The prevalence of CAC in big samples (*n* > 200) was 62% (95%CI 51–73%), while the summary prevalence in small samples (*n* < 200) was 59% (95%CI 49.3–69.2%), a significant difference was found in subgroup analysis (*p* < 0.05). A significant difference was found between cohort studies and cross-sectional studies 64% (95%CI, 54–74%) versus 59% (95%CI 50–55%) (*p* < 0.05). We found a significant difference in the estimated prevalence based on the year when the studies were published (before 2010 compared to 2010–2018).

We used meta-regression to explore the source of heterogeneity. We found that age (*r* = 1.009, *p* = 0.032) and dialysis duration (*r* = 1.005, *p* = 0.021) were positively associated with CAC prevalence, but there was no association between CAC prevalence and the proportion of men in the studies (*r* = 0.996, *p* = 0.136).

### All-cause mortality, cardiovascular mortality and cardiovascular events

**Figure 5.**
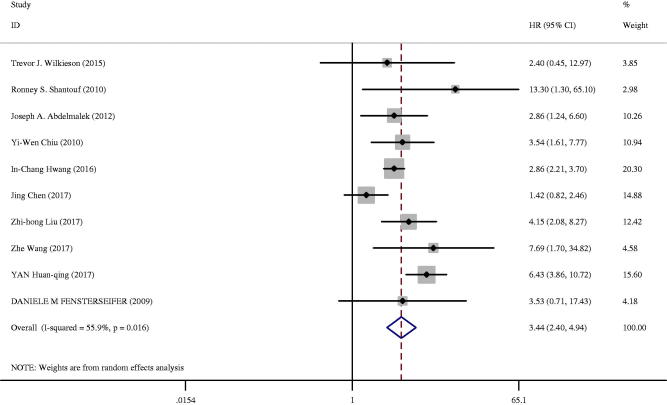
All-cause death among CKD patients in the highest versus lowest CAC score group.

Twelve studies analyzed the association between CAC and prognosis, among which three studies were also included in the studies of CAC prevalence. All-cause mortality events were investigated among 33 517 patients in 10 studies among the CKD patients [19,22,39,49,50,52,53,55–57], as shown in Table 2. Ten studies analyzed the risk of all-cause mortality in the highest versus lowest CAC score group (Figure 5) [19,22,39,49,50,52,53,55–57]. Two studies analyzed the risk of all-cause mortality with log-transformed CAC [22,50]. CAC was associated with an increased risk of all-cause mortality (HR 3.44; 95%CI: 2.40–4.94; *I^2^* = 55.9%; *p* = 0.016) in a random effect model in studies that measured HR (95%CI) in the highest CAC score group versus in the lowest CAC score group. In subgroup analysis, CKD stages significantly modified risk estimates for the association between CAC and mortality, with an increased risk among patients with ESRD receiving dialysis/kidney transplant compared with predialysis stages (HR 4.84 vs. 2.27; *p* = 0.018). There was no significant difference in all other subgroups, including country, age, proportion of men, diabetes, and hypertension. On performing meta-analysis, the CAC score on a natural log scale was related to an increased risk of all-cause mortality (HR 1.38; 95%CI 1.15–1.66; *I*^2^ = 68.4%, *p* = 0.042) in a random effect model.

**Figure 6.**
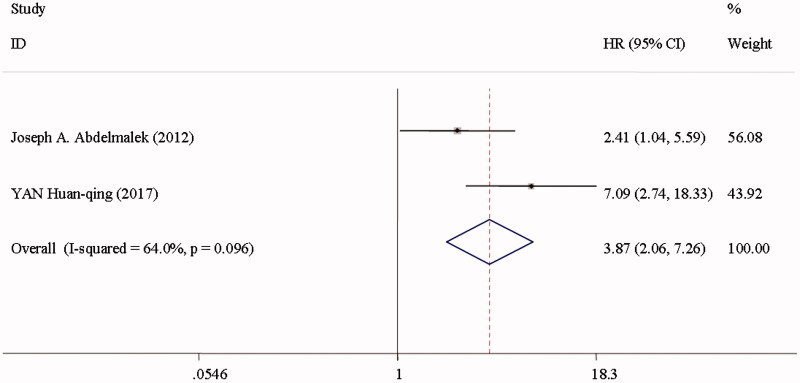
Cardiovascular death among CKD patients in the highest versus lowest CAC score group.

**Figure 7.**
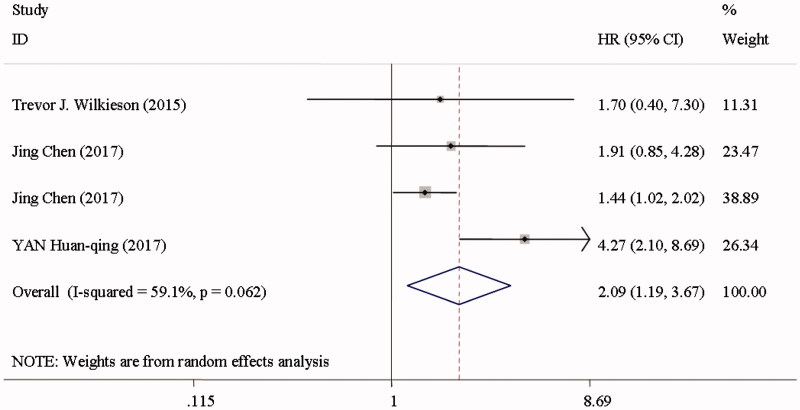
Cardiovascular events among CKD patients in the highest versus lowest CAC score group.

Cardiovascular mortality was reported in two studies among the CKD patients [19,22] (Figure 6). CVC was associated with a 2.87-fold greater risk of cardiovascular mortality (HR 3.87; 95%CI 2.06–7.26; *I*^2^ = 64%; *p* = 0.096) in a random effect model, and a 1.09-fold greater risk of cardiovascular events (HR 2.09; 95%CI 1.19–3.67; *I*^2^ = 59.1%; *p* = 0.062) (Figure 7) [22,49,54,55], when comparing individuals in the top with those in the bottom of CAC scores.

### Sensitivity analyses and publication bias

No significant reporting bias was observed among the 38 studies of CAC prevalence (Begg's test *p* = 0.513). Funnel plots were shown in Figure S1. There was no obvious bias of literature about CAC on mortality (Begg's test *p* = 0.592). Funnel plots were shown in Figure S2. We did not perform the evaluation for publication bias of literature about CAC on cardiovascular events, as the limited number of studies was included in the meta-analysis. The sensitivity analysis demonstrated that none of the articles significantly affected the pooled prevalence, prognosis.

## Discussion

Our meta-analysis of 38 selected studies investigated the prevalence Of CAC among patients with CKD. In our study, we found that CAC was highly prevalent among CKD patients, the pooled prevalence being 60% (95%CI, 53–68%).

According to our study, nearly two-thirds of CKD patients are diagnosed with CAC. Compared to studies on dialysis, there are far fewer studies about kidney transplant recipients. In consequence, sparse data and smaller sample size are partially responsible for the less precise estimated prevalence in the settings. Future studies are needed to explore the prevalence of CAC among renal transplant recipients. Due to the insufficient literature, CAC prevalence at different CKD stages could not be determined. Large clinical trials are needed to explore the prevalence of CAC at different CKD stages.

As higher heterogeneity was observed in the study, subgroup and meta-regression analyses were performed to detect the potential source of heterogeneity. Many studies have found the high prevalence of CAC in CKD patients across different regions and our meta-analysis validated these previously published results. Our analysis divided the studies based on region: Asian countries were compared with the rest of the world. This demonstrated that region was a potential source of heterogeneity. Subgroup analysis demonstrated that smaller sample sizes of these studies also contributed to the heterogeneity. The prevalence of studies published before 2010 is higher than that of the studies published 2010–2018. This implies that more attention is being paid to improve uremia factors such as CKD-mineral and bone disorder (MBD), and that treatment of CKD continues to improve. It was important that CAC prevalence was lower in cross-sectional studies, as well-conducted cohort studies provided a higher level of evidence than cross-sectional studies. Univariate meta-regression found that age and dialysis duration may be potential sources of heterogeneity, suggesting that older age was related to CAC [58]. In agreement with previous findings, we found that longer dialysis duration accelerated the progression of CAC [59].

The relationship between CKD and CAC prevalence is not fully understood, but several possible reasons are listed as follows. First, CKD-MBD is a common risk for CAC, particularly with higher plasma calcium and phosphate as well as severe secondary hyperparathyroidism. These higher levels are positively associated with vascular calcification. Second, calcification inhibitors are down-regulated in uremic factors [60]. Third, chronic inflammation is common among patients with CKD, and positively associated with CAC in CKD patients. Homocysteine and C-reactive protein (CRP) have been shown to up-regulate the expression of the inflammation process [61].

To our knowledge, this present meta-analysis is the first to explore prognostic role of CAC among CKD patients. We analyzed 12 studies, and found that CAC resulted in worse outcome. Mortality risk and cardiovascular events were higher among patients with coexisting CAC and CKD.

We found a low number of studies that analyzed cardiovascular events and mortality and only low to moderate heterogeneity was found; therefore, we did not conduct subgroup analysis and meta-analysis. Ten studies, reported the all-cause mortality risk (the highest CAC score group vs. the lowest CAC score group), were analyzed to detect the potential source of heterogeneity. In subgroup analysis, we found that patients receiving renal replacement therapy had higher all-cause mortality, and CKD stages were the source of heterogeneity. We did not demonstrate that other risk factors were responsible for the heterogeneity; the reasons may be smaller samples, limited data and short follow-up visits.

In CKD patients, calcification is found both in the intimal and medial layers of blood vessels. The mechanism of CAC that causes cardiovascular events and mortality is not fully understood. The possible reasons are listed as follows. Intimal calcification may lead to distal emboli, progressing to acute coronary syndromes [62]. Medial calcification induces stiffness of the coronary wall, resulting in reduction of myocardial perfusion. Medical calcification in the peripheral arteries increases myocardial afterload, causing left ventricular hypertrophy and cardiac failure [63,64].

Some limitations of our study have to be acknowledged. First, the results indicate considerable heterogeneity; the differences in study design, age, region, sample size, year of publication, CKD stages, and modality of dialysis were responsible for this variation. The short follow-up interval and the lower number of events recorded could affect the results, especially for cardiovascular and all-cause deaths. Second, some of the studies have small sample size, making it impossible to provide precise stratification according to CKD stages. Third, observational studies, depict associations, but do not establish cause and effect relationships. Finally, this article is not registered online to avoid repetition, which is one limitation of our study. However, we have kept searching the latest literature to track the progression of studies and avoid duplication of effort during the study period.

## Conclusions

CAC is prevalent in patients with CKD. Severe CAC significantly and rapidly deteriorates the prognosis among CKD patients, and CAC significantly affects cardiovascular and all-cause mortality. Future large prospective studies are necessary to explore CAC prevalence at different stages of CKD. Moreover, long-term follow-up visits are necessary to understand the effect of CAC on patients at different CKD stages. Future researches should explore the possible mechanism and therapeutic interventions to halt CAC.

## Supplementary Material

Supplemental Material
