# Supplementary material for: Prevalence of coronary artery calcification and its association with mortality, cardiovascular events in patients with chronic kidney disease: a systematic review and meta-analysis
Source: Ren Fail. 2019 Apr 24;41(1):244–56. doi: 10.1080/0886022X.2019.1595646 (PMC6493287; doi:10.1080/0886022X.2019.1595646)
Supplement: Supplemental Material [file IRNF_A_1595646_SM8387.zip › CAC search appendix1 .pdf]

Search appendix 1

PubMed:

1. (coronary artery calcification [MeSH Terms]) OR coronary artery calcification [Text Word]
2. (vascular calcification [MeSH Terms]) OR vascular calcification [Text Word]
3. calcif*[Text Word]
4. (CAC[Abstract]) OR CAC[Title]
5. (((((coronary artery calcification[MeSH Terms]) OR coronary artery calcification[Text Word])) OR ((vascular calcification[MeSH Terms]) OR vascular calcification[Text Word])) OR calcif*[Text Word]) OR ((CAC[Abstract]) OR CAC[Title])
6. (chronic kidney[Text Word]) OR chronic kidney[Text Word]
7. (Kidney Failure) OR renal failure
8. (Uremia[Title]) OR Uremia[Abstract]
9. (Kidney disease [MeSH Terms]) OR kidney disease [Text Word]
10. (((CKF [Text Word]) OR CKD [Text Word]) OR CRF [Text Word]) OR CRD[Text Word]
11. (((((((chronic kidney[Text Word]) OR chronic kidney[Text Word])) OR ((Kidney Failure) OR renal failure)) OR ((Uremia[Title]) OR Uremia[Abstract])) OR ((kidney disease[MeSH Terms]) OR kidney disease[Text Word])) OR ((((CKF[Text Word]) OR CKD[Text Word]) OR CRF[Text Word]) OR CRD[Text Word]))
12. (death [MeSH Terms]) OR death[Text Word]
13. (mortality[MeSH Terms]) OR mortality[Text Word]
14. (((mortality[MeSH Terms]) OR mortality[Text Word])) OR ((death[MeSH Terms]) OR death[Text Word])
15. Search (((((((((chronic kidney[Text Word]) OR chronic kidney[Text Word])) OR ((Kidney Failure) OR renal ailure)) OR ((Uremia[Title]) OR Uremia[Abstract])) OR ((kidney disease[MeSH Terms]) OR kidney disease[Text Word])) AND ((((CKF[Text Word]) OR CKD[Text Word]) OR CRF[Text Word]) OR CRD[Text Word]))) AND ((((mortality[MeSH Terms]) OR mortality[Text Word])) OR ((death[MeSH Terms]) OR death[Text Word]))) AND ((((((coronary artery calcification[MeSH Terms]) OR coronary artery calcification[Text Word])) OR ((vascular calcification[MeSH Terms]) OR vascular calcification[Text Word])) OR calcif*[Text Word]) OR ((CAC[Abstract]) OR CAC[Title]))

Embase:

#1 coronary AND ('artery' OR 'artery'/exp OR artery) AND ('calcification' OR 'calcification'/exp OR calcification)
#2 vascular AND ('calcification' OR 'calcification'/exp OR calcification)
#3 'cac':ab,ti
#4 'calcif*':ab,ti
#5 #1 OR #2 OR #3 OR #4
#6 chronic AND ('kidney' OR 'kidney'/exp OR kidney) AND ('disease' OR 'disease'/exp OR disease)
#7 'kidney disease'/exp OR 'kidney disease'
#8 'kidney failure'/exp OR 'kidney failure'
#9 'chronic kidney' OR 'chronic renal'

#10 ckf OR ckd OR 'crf'/exp OR 'crf' OR crd
#11 'uremia'/exp OR 'uremia'
#12 'hemodialysis' OR 'hemodialysis'/exp OR hemodialysis
#13 peritoneal AND ('dialysis' OR 'dialysis'/exp OR dialysis)
#14 'dialysis':ab,ti
#15 #6 OR #7 OR #8 OR #9 OR #10 OR #11 OR #12 OR #13 OR #14
#16 'death'/exp OR 'death'
#17 'mortality'/exp OR 'mortality'
#18 #16 OR #17
#19 #5 AND #15 AND #18
#20 #5 AND #15 AND #18 AND [humans]/lim

Web of Science
#1 TS= “coronary artery calcification”
#2 TS= “vascular calcification”
#3 TS= “CAC”
#4 #1 OR #2 OR #3
#5 TS= “chronic kidney disease”
#6 TS= “chronic renal failure”
#7 TS= “chronic kidney failure”
#8 TS= “hemodialysis”
#9 TS= “peritoneal dialysis”
#10 TS= “uremia”
#11 #5 OR #6 OR #7 OR #8 OR #9 OR #10
#12 TS= “death”
#13 TS= “mortality”
#14 #12 OR #13
#15 #4 AND #11 AND #14
