# Supplementary material for: Prevalence of coronary artery calcification and its association with mortality, cardiovascular events in patients with chronic kidney disease: a systematic review and meta-analysis
Source: Ren Fail. 2019 Apr 24;41(1):244–56. doi: 10.1080/0886022X.2019.1595646 (PMC6493287; doi:10.1080/0886022X.2019.1595646)
Supplement: Supplemental Material [file IRNF_A_1595646_SM8387.zip › Funnel plots.pdf]

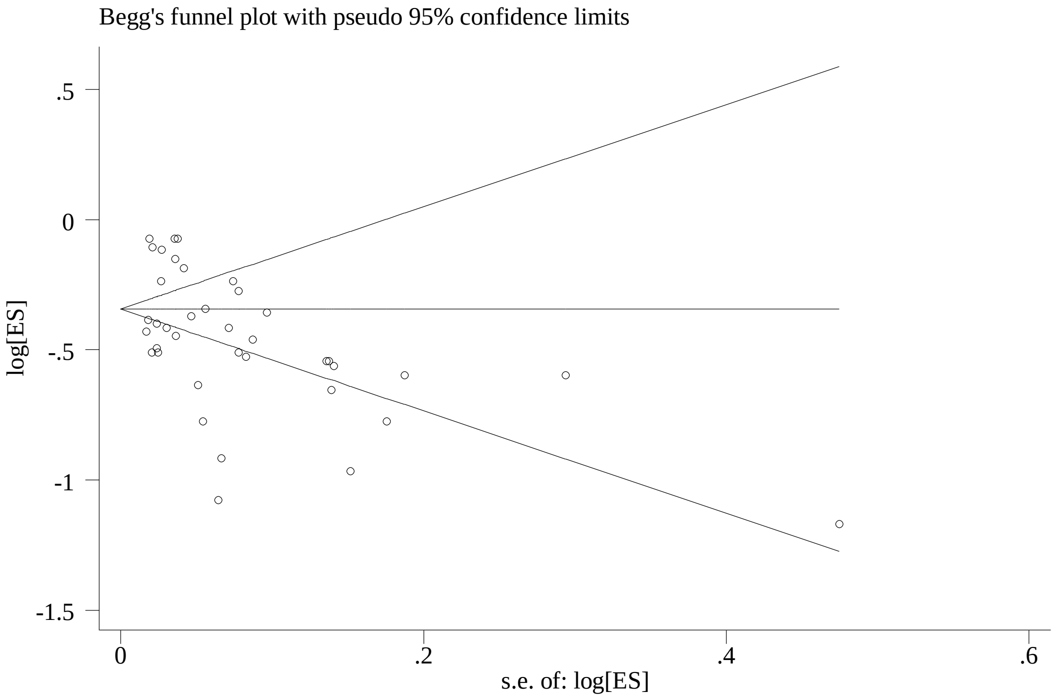

Figure S1 Funnel plot to assess bias of CAC prevalence

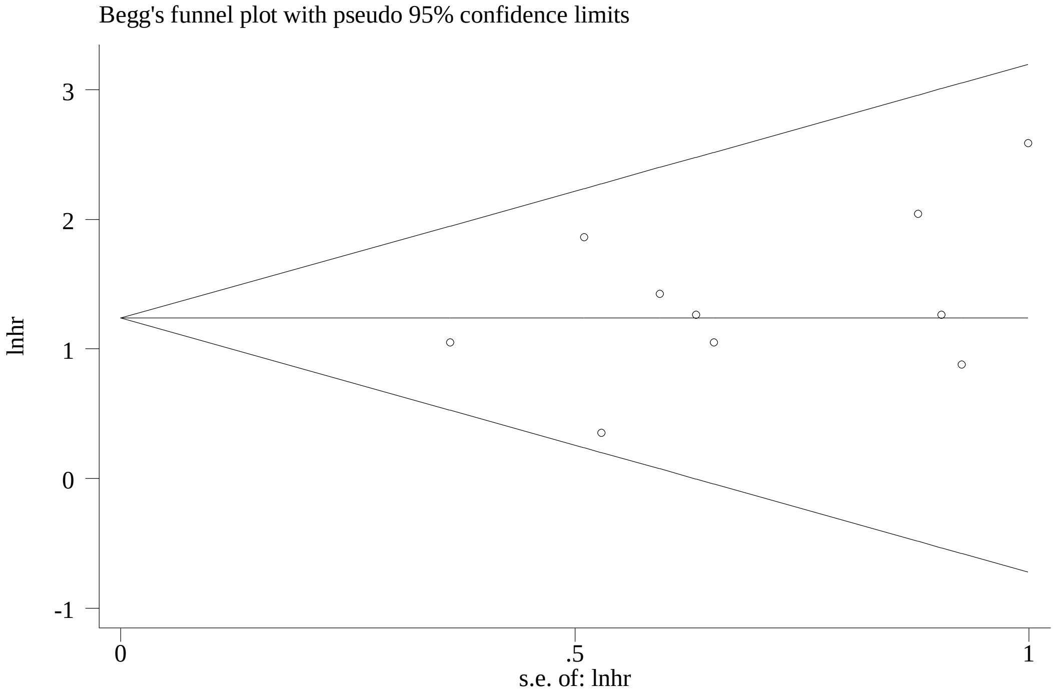

Figure S2 Funnel plot to assess bias in estimates of CAC on mortality
